# The association between perceived mortality exposure and nurses’ depressive symptomes, through life satisfaction, and sleep quality

**DOI:** 10.3389/fpubh.2026.1872055

**Published:** 2026-07-16

**Authors:** Rawaih Falatah, Norah Al Sharaef, Reem Al-Dossary, Majed Alamri, Jalal Alharbi, Khalid Aljohani, Hamdan Albaqawi, Bader Alrasheadi, Noura Almadani, Mohammed Aljohani

**Affiliations:** 1 Nursing Administration and Education Department, College of Nursing, King Saud University, Riyadh, Saudi Arabia; 2 Ministry of Health, Prince Faisal Bin Khalid Cardiac Center, Abha, Saudi Arabia; 3 Nursing Education Department, Nursing College, Imam Abdulrahman Bin Faisal University, Dammam, Saudi Arabia; 4 Department of Medical, Surgical and Critical Care Nursing, College of Nursing, University of Hafr Al Batin, Hafr Al Batin, Saudi Arabia; 5 Community Health Nursing Department, Nursing College Taibah University, Al-Madinah, Saudi Arabia; 6 Medical Surgical Nursing Department, College of Nursing, University of Hail, Hail, Saudi Arabia; 7 Department of Nursing Administration, College of Nursing, Majmaah University, Majmaah, Saudi Arabia; 8 Community and Psychiatric Mental Health Nursing Department, Nursing College, Princess Nourah Bint Abdulrahman University, Riyadh, Saudi Arabia; 9 Medical-Surgical Nursing Department, Nursing College, Taibah University, Al-Madinah, Saudi Arabia

**Keywords:** coping, depression, life satisfaction, mortality, nurses, sleep quality, stress

## Abstract

**Introduction:**

Nurses working in high-mortality clinical settings are frequently exposed to patient suffering and death. This might contribute to occupational stress and adverse psychological outcomes. However, the mechanisms linking this exposure to depressive symptoms remain unclear. This study examines the association between perceived mortality exposure and depressive symptoms and the mediating roles of life satisfaction and sleep quality.

**Methods:**

A cross-sectional study was conducted among 200 nurses using an online survey. Data were analyzed using correlation, regression, and mediation analysis with Hayes’ PROCESS macro.

**Results:**

Perceived mortality exposure was significantly associated with higher depressive symptoms and poorer sleep quality. Life satisfaction was inversely associated with depressive symptoms, whereas sleep quality was not. Nonetheless, the overall regression model, including perceived mortality exposure and life satisfaction, explained only a modest proportion of the variance in depressive symptoms. The mediation analysis showed no significant indirect effects through life satisfaction or sleep quality.

**Discussion:**

Perceived mortality exposure represents a potentially significant occupational stressor for nurses, but the lack of mediation indicates that factors beyond life satisfaction and sleep quality may better explain its impact on depressive symptoms.

## 1 Introduction

Nurses working in high-mortality clinical settings are routinely exposed to the suffering and death of patients. These experiences can evoke strong emotional reactions and chronic occupational stress (1, 2). In the literature, continuous exposure to patient death has been linked to compassion fatigue, emotional exhaustion, and symptoms of depression (3, 4). According to the Transactional Model of Stress and Coping (5), stress can arise when environmental demands exceed an individual’s perceived coping resources. In such contexts, nurses might perceive frequent patient deaths as personally and professionally threatening. This perception may be amplified when the nurses lack adequate emotional or institutional support to process loss. These stress appraisals can manifest as physiological disturbances (e.g., poor sleep) (6) and psychological strain (e.g., lower life satisfaction and depressive symptoms) (7).

Life satisfaction represents the psychological component of subjective well-being. It reflects an individual’s evaluation of the overall quality of life (8). When nurses experience persistent emotional strain and inadequate recovery, they are more likely to perceive their lives as less satisfying. Furthermore, frequent exposure to critically ill patients and patient deaths is associated with stress and lower life satisfaction among nurses (9). Hence, nurses with lower life satisfaction might experience poor sleep quality. Importantly, reduced life satisfaction might be accompanied by psychological distress, which can interfere with sleep patterns and recovery processes (2). This suggests that sleep quality may be a key mechanism through which life satisfaction influences overall well-being. Therefore, examining sleep quality is essential to better understand how diminished life satisfaction translates into adverse health outcomes among nurses (10, 11).

Generally, nurses report lower sleep quality compared to other professionals. In particular, nurses who work in critical areas are considered to have lower sleep quality (3). Sleep disturbances are also common among nurses due to factors such as shift work, long working hours, and occupational stress. In particular, rotating and night shifts can disrupt circadian rhythms and negatively affect sleep quality (12). Within the nursing profession, poor sleep quality is associated with reduced work performance, increased medical errors, and lower quality of life (13–15). Moreover, sleep disturbance is included among the diagnostic symptoms of depression. However, sleep quality is also recognized as a distinct construct that influences physical health, cognitive functioning, occupational performance, and psychological well-being. Research suggests that poor sleep quality may both contribute to and result from psychological distress, highlighting its importance as a separate factor in studies examining mental health outcomes among nurses (12). Thus, it can be stated that poor sleep quality has been associated in the literature with higher levels of depressive symptoms among nurses (10, 11).

Depressive symptoms are deemed to be among the most common psychological outcomes of prolonged occupational stress (16, 17). Empirical evidence indicates that depression is associated with sleep deprivation through heightened cognitive and emotional arousal. Indeed, sleep disruption has been shown to diminish emotional stability, concentration, and life satisfaction (18, 19). Consequently, life satisfaction and sleep disturbance may act as sequential mediators through which high mortality exposure increases nurses’ risk for depression. This study aims to understand the association between perceived mortality exposure and depressive symptoms among nurses through life satisfaction and sleep quality. Understanding this interplay might help in developing organizational strategies that support nurses in high-stress, high-mortality environments.

This study was guided by concepts from the Transactional Model of Stress and Coping (5). The model conceptualizes stress as a dynamic interaction between individuals and their environment. According to this model, stress arises when perceived environmental demands exceed available coping resources. Individuals engage in primary appraisal, where an event is evaluated as threatening, challenging, or benign, and secondary appraisal, where coping resources are assessed. Importantly, psychological outcomes are not determined solely by the stressor itself but by how individuals interpret and respond to it (5).

Within the nursing context, perceived mortality exposure represents a significant environmental stressor, particularly in high-acuity settings where exposure to patient death is frequent. Nurses cognitively appraise repeated exposure to mortality as emotionally demanding, potentially overwhelming their coping capacity. When coping resources, such as emotional resilience, social support, and adequate rest, are insufficient, this stress may manifest as depressive symptoms. However, the model suggests that this relationship is not direct; rather, it is shaped by intermediate psychological and physiological processes.

Guided by selected concepts from this framework, the present study conceptualizes life satisfaction and sleep quality as key mediating mechanisms linking nurses’ perception of mortality exposure to depressive symptoms. Specifically, higher perceived mortality exposure may negatively influence nurses’ life satisfaction, reflecting diminished subjective well-being, and may also disrupt sleep quality as a physiological response to stress. In turn, reduced life satisfaction and poor sleep quality are expected to increase vulnerability to depressive symptoms. Thus, the model posits that perceived mortality exposure influences depressive symptoms indirectly through its impact on life satisfaction and sleep quality, highlighting the role of cognitive appraisal and recovery processes in shaping psychological outcomes among nurses.

This study aims to examine the direct and indirect relationship between perceived mortality exposure and depressive symptoms among nurses, through the roles of life satisfaction and sleep quality. The hypotheses of this study are:

*H1:* Perceived mortality exposure is significantly associated with nurses’ depressive symptoms.

*H2:* Nurses’ life satisfaction and sleep quality mediate the association between perceived mortality exposure and nurses’ depressive symptoms.

## 2 Methods

### 2.1 Study design

This study utilized a cross-sectional correlational design.

### 2.2 Study setting and sample

The study was conducted in a medical city in central Saudi Arabia. This medical city is considered one of the largest in the Middle East. Its clinical capacity is 1,095 beds, with a nursing workforce exceeding 2,800 nurses. The medical city provides adult, women’s, and pediatric services.

Convenience sampling was used in this study. The required sample size was estimated using G*Power version 3.1.9.6. An *a priori* power analysis for linear multiple regression was conducted using a medium effect size (f^2^ = 0.15), an alpha level of 0.05, and a statistical power of 0.80 (20); the estimated sample size was 146 participants. The inclusion criteria included registered nurses who had been employed in the medical city for at least one year, provided direct patient care, and were willing to provide informed consent. The exclusion criteria included nurses in administrative positions. Eligible nurses were recruited from various inpatient and outpatient units within the medical city using a convenience sampling approach. The survey link was distributed electronically through nursing managers and professional nursing communication channels to 250 eligible nurses. Of these, 200 completed the survey and were included in the final analysis. The remaining 50 nurses did not respond to the survey invitation, resulting in an 80% response rate.

### 2.3 Measurement and scoring

#### 2.3.1 Sociodemographic characteristics

A self-administered demographic questionnaire was used to collect information about participants’ personal and professional characteristics. The variables included age, gender, nationality, marital status, presence of chronic illness, general health status, family caregiving responsibilities, education level, monthly income, years of experience in nursing, work unit, and years of experience in the current institution. Prior training in mental health or end-of-life care was measured using two dichotomous items (yes/no): (1) participation in a training program in mental health, and (2) participation in a training program in coping with patient death. Participants who answered *yes* specified the duration of the training program.

#### 2.3.2 Perceived mortality exposure

Perceived mortality exposure was conceptualized as nurses’ perceived exposure to patient deaths, regardless of the specific clinical setting in which they worked. It was assessed using five items developed for this study. The items capture both the general and temporal aspects of perceived mortality exposure in the clinical unit. The first item measured the overall perceived mortality rate (“How would you consider the mortality rate in your unit?”), rated on a three-point Likert type scale (1 = *low*, 2 = *average*, 3 = *high*). Four additional items assessed the perceived mortality exposure in the past week, month, six months, and year, respectively, using open-ended numeric responses (e.g., number of deaths). These items were designed to provide both cross-sectional and longitudinal indicators of exposure to patient deaths. For analysis, mortality exposure was treated both as a categorical variable (low, average, high) and as a continuous exposure index derived from the temporal frequency items.

#### 2.3.3 Satisfaction with life scale (SWLS)

Overall life satisfaction was measured using the Satisfaction With Life Scale (SWLS) developed by Diener et al. (8). It consists of five self-report items that measure global cognitive evaluations of one’s overall satisfaction with life (e.g., “In most ways my life is close to my ideal”). Each item is rated on a seven-point Likert-type scale ranging from 1 (*strongly disagree*) to 7 (*strongly agree*). All items are positively worded and summed to yield a total score ranging from 5 to 35, with higher scores indicating greater life satisfaction. The SWLS demonstrates excellent internal consistency (*α* ≈ 0.87) (8). In the present study, the total score was calculated as the sum of the five items, and internal consistency reliability was evaluated using Cronbach’s α (0.88).

#### 2.3.4 Pittsburgh sleep quality index (PSQI)

Sleep quality and disturbances over the past month were measured using the Pittsburgh Sleep Quality Index (21). The PSQI contains 19 self-rated items grouped into seven subscales that evaluate different aspects of sleep: (a) subjective sleep quality, (b) sleep latency, (c) sleep duration, (d) habitual sleep efficiency, (e) sleep disturbances, (f) use of sleep medication, and (g) daytime dysfunction. Each subscale is scored from 0 (no difficulty) to 3 (severe difficulty). The seven subscale scores are summed to obtain a global sleep quality score, which ranges from 0 to 21, with higher scores indicating poorer sleep quality. According to the original scoring guidelines, a global PSQI score greater than 5 indicates poor sleep quality, whereas a score of 5 or less indicates good sleep quality (19). The PSQI has demonstrated acceptable reliability in diverse populations (*α* = 0.70–0.83) (6, 21, 22). In the current study, Cronbach’s α was 0.75.

#### 2.3.5 Zung self-rating depression scale (SDS)

Depressive symptoms were assessed using the Zung Self-Rating Depression Scale (23), a 20-item self-report instrument measuring affective, psychological, and somatic aspects of depression (e.g., “I feel down-hearted and blue”). Each item is rated on a four-point Likert-type scale reflecting the frequency of symptoms during the past week (1 = *a little of the time*, 4 = *most of the time*). Ten positively worded items were reverse-scored to ensure directional consistency. The total raw score was calculated by summing all 20 items (range = 20–80), with higher scores indicating greater depressive symptomatology. A standardized index score was then derived by multiplying the raw score by 1.25, yielding a range of 25–100. Scores were classified as follows: < 50 = normal, 50–59 = mild, 60–69 = moderate, and ≥ 70 = severe depression. The SDS has demonstrated high internal consistency in previous studies [*α* = 0.80–0.90; Zung (23)] (24, 25). In the current study, Cronbach’s α was 0.78, which indicates acceptable reliability.

### 2.4 Ethical consideration

This study was conducted in accordance with the ethical principles of the Declaration of Helsinki. Ethical approval was obtained from the King Saud University Institutional Review Board (E-24-9127) and the Riyadh Second Health Cluster (H-01-012). Participants were informed in the invitation message about the purpose of the study, the voluntary nature of their participation, the expected time to complete the survey, and that the data would be anonymous. Online informed consent was obtained from all participants before starting the online survey. Participants who answered “no” to the consent question were directed to the end of the survey, where their desire not to participate was acknowledged, and they were thanked for their time.

### 2.5 Data collection

After obtaining IRB approval, the study survey items were entered into Google Forms, with the informed consent form presented on the first page. To facilitate access to nurses working in clinical departments, the researcher communicated with the nursing office at the medical city, explained the purpose of the study, and provided an estimate of the time required to complete the survey. The link was then distributed to the eligible nurses via their email addresses.

### 2.6 Data analysis

Data were analyzed using the Statistical Package for the Social Sciences (SPSS), version 30.0. Descriptive statistics, including frequencies, percentages, means, and standard deviations, were used to summarize participants’ demographic characteristics and study variables. The normality of continuous variables was assessed using skewness and kurtosis values. Independent samples t-tests and one-way ANOVA were used to examine differences in life satisfaction, sleep quality, and depressive symptoms across sociodemographic characteristics. Pearson’s correlation coefficients were used to assess relationships among key study variables. Hierarchical multiple regression analyses were performed to examine the predictive effects of demographic variables (years of nursing experience and nationality) and perceived mortality exposure on life satisfaction, sleep quality, and depressive symptoms. Mediation analysis was conducted using Hayes’ PROCESS macro (Model 6) to examine whether life satisfaction and sleep quality mediated the relationship between mortality exposure and depressive symptoms among nurses. Bootstrapping with 5,000 resamples was used to estimate indirect effects, and 95% confidence intervals were applied; mediation was considered significant if the confidence interval did not include zero. Statistical significance was set at *p* < 0.05.

## 3 Results

### 3.1 Descriptive analysis

A total of 200 nurses participated in the study (Table 1). The majority were female (77.5%) and non-Saudi (78.5%). In terms of marital status, 59.5% were married. Most participants (87%) reported no chronic illness. When asked to rate their general health, 18.0% described it as excellent, while 12.5 and 0.5% reported their health as acceptable and poor, respectively. Over half of the respondents (56%) reported having a family caregiver role. The majority held a bachelor’s degree (85%). Regarding previous training, 15% of participants had received training in the field of mental health, and 12% had received training in dealing with patient death. In terms of workplace perceived mortality exposure, most participants reported low perceived mortality exposure in their area (72.5%), followed by average (25.0%) and high (2.5%).

**Table 1:** Descriptive statistics of categorical variables (*n* = 200).

Variables		Frequency (%)
Gender	Male	45 (22.5)
	Female	77.5 (77.5)
Nationality	Saudi	43 (21.5)
	Non-Saudi	157 (78.5)
Marital Status	Single	75 (37.5)
	Married	119 (59.5)
	Divorced	1 (0.5)
	Widowed	1 (0.5)
	Separated	4 (2.0)
Chronic illness	Yes	26 (13)
	No	174 (87)
General health rating	Poor	1 (0.5)
	Acceptable	25 (12.5)
	Good	78 (39.0)
	Very good	60 (30)
	Excellent	36 (18)
Family caregiver role	Yes	112 (56)
	No	88 (44)
Level of education	Diploma	14 (7.0)
	Bachelor’s	170 (85.0)
	Master’s	16 (8.0)
Work settings *i*	Adult medical surgical units	20 (10.0)
	Emergency	7 (3.5)
	Intensive care unit	38 (19.0)
	Obstetrics and Gynecology	23 (11.5)
	Operating room	15 (7.5)
	Outpatient department	8 (4.0)
	Rehabilitation	39 (19.5)
	Oncology	36 (18.0)
	Cardiac	2 (1.0)
	Radiology and endoscopy	2 (1.0)
	Others	10 (5.0)
Training in the field of mental health	Yes	30 (15.0)
	No	170 (85.0)
Training in adapting to patient death	Yes	24 (12.0)
	No	176 (88.0)
Perceived mortality exposure	Low	145 (72.5)
	Average	50 (25.0)
	High	5 (2.5)
Satisfaction with life scale	Extremely dissatisfied	1 (0.5)
	Dissatisfied	13 (6.5)
	Slightly dissatisfied	17 (8.5)
	Neutral	5 (2.5)
	Slightly satisfied	41 (20.5)
	Satisfied	89 (44.5)
	Extremely satisfied	34 (17.0)
Sleep efficiency	Very good (≥85%)	131 (65.8)
	Fairly good (75–84%)	30 (15.1)
	Fairly bad (65–74%)	21 (10.6)
	Very bad (<65%)	17 (8.5)
Sleep disturbance	None	85 (42.5)
	Moderate	23 (11.5)
	Severe	92 (46.0)
Use of sleep medication	None	13 (6.5)
	< Once a week	143 (71.5)
	1–2 times a week	26 (13.0)
	≥3 times a week	18 (9.0)
Daytime dysfunction	None	8 (4.0)
	Mild	28 (14.0)
	Moderate	92 (46.0)
	Severe	72 (36.0)
Minutes to fall asleep	≤15 min	34 (17.0)
	16–30 min	47 (23.5)
	31–60 min	21 (10.5)
	>60 min	98 (49.5)
Overall sleep quality	Good	2 (1.0)
	Poor	198 (99.0)
Depressive symptoms severity	Normal	73 (36.5)
	Mild	69 (34.5)
	Moderate	44 (22.0)
	Severe	14 (7.0)

Regarding life satisfaction, about 16% of participants reported dissatisfaction with their lives. In terms of sleep characteristics, approximately 20% reported poor sleep efficiency, and nearly half (46.0%) experienced severe sleep disturbance. Regarding the use of sleep medication, 71.5% reported using it less than once per week. For daytime dysfunction, 46.0% reported a moderate level. When asked about the time required to fall asleep, nearly half (49.5%) took more than 60 min to fall asleep. Overall, 99.0% of participants were classified as having poor sleep quality according to the established PSQI cut-off score (>5). Although this prevalence appears high, participants also reported substantial sleep-related difficulties across multiple PSQI domains, including prolonged sleep latency, frequent sleep disturbances, and moderate-to-severe daytime dysfunction. Finally, regarding depressive symptoms severity, 36.5% were classified as normal, while 7.0% reported a severe level of depressive symptoms (Table 1).

Table 2 shows the descriptive statistics for the continuous variables. The mean age of the participants was 37.32 years (SD = 8.63), ranging from 22 to 59 years. The participants reported an average monthly income of SR 8994.65 (SD = 3436.00). The mean nursing experience was 12.59 years (SD = 7.97), and the mean organizational experience was 6.27 years (SD = 5.43). Regarding patient outcomes, the average perceived mortality exposure within six months was 1.69 (SD = 2.60), and within one year was 3.17 (SD = 4.74). The mean time in bed was 6.60 h (SD = 2.35).

**Table 2:** Descriptive statistics for the continuous variables (*n* = 200).

Variables	Mean (SD)	Range (maxi-mini)	Skewness		Kurtosis	
			Statistic	Std. error	Statistic	Std. error
Age	37.32 (8.63)	37 (59–22)	0.470	0.172	−0.422	0.342
Monthly income	8994.65 (3436.00)	16,500 (20000–3,500)	1.216	0.172	1.40	0.342
Nursing experience	12.59 (7.97)	36 (37–1)	0.753	0.172	0245	0.342
Organizational experience	6.27 (5.43)	20 (21–1)	1.127	0.172	0.181	0.342
Average perceived mortality exposure in six months	1.69 (2.604)	13 (13–0)	2.017	0.172	3.809	0.342
Average perceived mortality exposure in one year	3.17 (4.735)	24 (24–0)	2.305	0.172	4.922	0.342
Time in bed (hours)	6.60 (2.354)	23 (23–0)	3.068	0.172	17.27	0.342
SWLS	25.68 (5.966)	26 (35–9)	−0.824	0.172	0.104	0.342
SDS	42.565 (8.729)	48 (70–22)	0.016	0.172	−0.059	0.342
PSQI	10.38 (2.352)	12 (16–4)	−0.011	0.172	−0.408	0.342

In terms of the study variables, the mean score on the Satisfaction with Life Scale (SWLS) was 25.68 (SD = 5.97), indicating a moderate to high level of life satisfaction. The Pittsburgh Sleep Quality Index (PSQI) mean score was 10.38 (SD = 2.35), which reflects generally poor sleep quality in the sample (scores >5 indicate poor sleep). The Self-Rating Depression Scale (SDS) mean score was 42.57 (SD = 8.73), suggesting mild to moderate depressive symptoms among the participants.

### 3.2 Mean difference in the study variables based on the sociodemographics

Two-tailed independent-samples *t*-tests were conducted to examine differences in life satisfaction, sleep quality, and depressive symptoms based on participants’ gender, nationality, and chronic illness status. Results showed no significant mean differences in the study variables based on participant gender or chronic illness. However. results indicated a significant difference in life satisfaction (*t* = −2.293, *p* < 0.01) and depressive symptoms (*t* = 2.512, *p* < 0.01) across nationalities. Specifically, Saudi nurses reported lower life satisfaction (M = 23.58, SD = 7.08) and higher levels of depressive symptoms (M = 54.59, SD = 9.78) compared to non-Saudi nurses (SWLS: M = 26.25, SD = 5.5; SDS: M = 41.76, SD = 8.27). No significant difference was found in sleep quality (*t* = 1.015, *p* > 0.05), suggesting that participants from both nationality groups experienced comparable levels of sleep quality.

One-way ANOVA analyses were conducted to examine the mean differences in the study variables across chronic illness diagnoses, education level, and family caregiving status. The only significant difference was in the overall sleep quality score, *F*(7–192) = 2.15, *p* = 0.04, with lower sleep quality associated with a diagnosis of diabetes. Additionally, one-way ANOVA indicated no significant differences in perceived mortality exposure, life satisfaction, sleep quality, or depressive symptoms across work settings (all *p* > 0.05).

### 3.3 Correlational analysis

Pearson’s correlation coefficients were computed to examine the relationships among sociodemographic characteristics and study variables. The results are presented in Table 3. As shown, age was significantly and negatively correlated with SDS (*r* = −0.139, *p* = 0.049). Nursing experience (*r* = −0.192, *p* = 0.006) and organizational experience (*r* = −0.194, *p* = 0.006) were negatively correlated with SDS. These findings suggest that older and more experienced nurses reported fewer depressive symptoms. Life satisfaction also showed a weak positive correlation with organizational experience (*r* = 0.144, *p* = 0.042). On the other hand, nurses’ income was not significantly correlated with any of the study variables.

**Table 3:** Correlation.

Variables	1	2	3	4	5	6	7
1. Age							
2. Income	0.198**						
3. Nursing experience	0.858***	0.215**					
4. Organizational experience	0.599***	0.160*	0.691***				
5. Perceived mortality exposure	−0.053	−0.059	−0.053	−0.057			
6. SWLS	−0.016	−0.015	0.035	0.144*	−0.074		
7. PSQI	−0.073	0.029	−0.083	−0.016	0.251***	−0.083	
8. SDS	−0.139*	−0.031	−0.192**	−0.194**	0.162*	−0.448***	0.102

*p < 0.05. **p < 0.01. ***p < 0.001 (two-tailed).

Among the study variables, a significant negative correlation was found between life satisfaction (SWLS) and depressive symptoms (SDS) (*r* = −0.448, *p* < 0.001), indicating that higher life satisfaction was associated with lower depressive symptoms. Perceived mortality exposure was positively correlated with Sleep quality (PSQI) (*r* = 0.251, *p* < 0.001) and SDS (*r* = 0.162, *p* = 0.0220), suggesting that higher perceived exposure to patient mortality was associated with poorer sleep quality and higher depressive symptoms. However, PSQI was not significantly related to SDS.

### 3.4 Results based on the study hypothesis

*H1:* Perceived mortality exposure is significantly associated with nurses’ depressive symptoms.

Hierarchical regression analysis was conducted to examine the association between perceived mortality exposure and depressive symptoms while controlling for demographic variables. Age was excluded from the model due to its high correlation with years of nursing experience (r = 0.858), indicating potential multicollinearity. Years of nursing experience were retained as they were more theoretically relevant to nurses’ clinical exposure and professional development. In Model 1, which included years of nursing experience and nationality, the overall model was significant, R^2^ = 0.050, *F*(2, 197) = 5.16, *p* = 0.007, explaining 5.0% of the variance in depressive symptoms. Years of nursing experience were negatively associated with depressive symptoms (B = −0.162, *p* = 0.049), whereas nationality was not a significant predictor (see Table 4).

**Table 4:** Mediation analysis.

Path	Effect (B)	SE/BootSE	t	*p*	95% CI (LL, UL)
Total effect (c) Perceived mortality exposure → SDS	1.67	0.70	2.37	0.019	[0.28, 3.06]
Direct effect (c′) Perceived mortality exposure → SDS	1.23	0.66	1.86	0.064	[−0.07, 2.54]
Direct paths (a and b paths)					
a₁: Perceived mortality exposure → SWLS	−0.62	0.49	−1.26	0.210	[−1.58, 0.35]
a₂: Perceived mortality exposure → PSQI	0.68	0.19	3.57	< 0.001	[0.30, 1.06]
b₁: SWLS → SDS	−0.62	0.09	−6.64	< 0.001	[−0.80, −0.44]
b₂: PSQI → SDS	0.08	0.24	0.32	0.746	[−0.40, 0.55]
Indirect effects (a × b)					
a₁b₁: Perceived mortality exposure → SWLS → SDS	0.38	0.25	—	—	[−0.05, 0.95]
a₂b₂: Perceived mortality exposure → PSQI → SDS	0.05	0.19	—	—	[−0.33, 0.44]
a₁d₂₁b₂: Perceived mortality exposure → SWLS → PSQI → SDS	0.001	0.007	—	—	[−0.01, 0.02]
Total indirect effect	0.44	0.32	—	—	[−0.15, 1.11]

a paths indicate the relationships between perceived mortality exposure and the mediators, whereas b paths indicate the relationships between the mediators and depressive symptoms. c denotes the total effect of perceived mortality exposure on depressive symptoms, and c′ denotes the direct effect after adjustment for the mediators.

In Model 2, after adding perceived mortality exposure, the overall model remained significant, R^2^ = 0.076, *F*(3, 196) = 5.38, *p* = 0.001, explaining 7.6% of the variance in depressive symptoms. The addition of perceived mortality exposure resulted in a significant increase in explained variance (ΔR^2^ = 0.026, F-change (1, 196) = 5.60, *p* = 0.019). Perceived mortality exposure emerged as a significant positive predictor of depressive symptoms (B = 1.667, SE = 0.705, *β* = 0.163, p = 0.019, 95% CI [0.278, 3.057]). Years of nursing experience and nationality were no longer significant predictors in the final model. Although perceived mortality exposure contributed significantly to the model, the relatively small proportion of explained variance suggests that depressive symptoms are influenced by multiple factors beyond those examined in the present study.

*H2:* Nurses’ life satisfaction and sleep quality mediate the association between perceived mortality exposure and nurses’ depressive symptoms.

**Figure 1:**
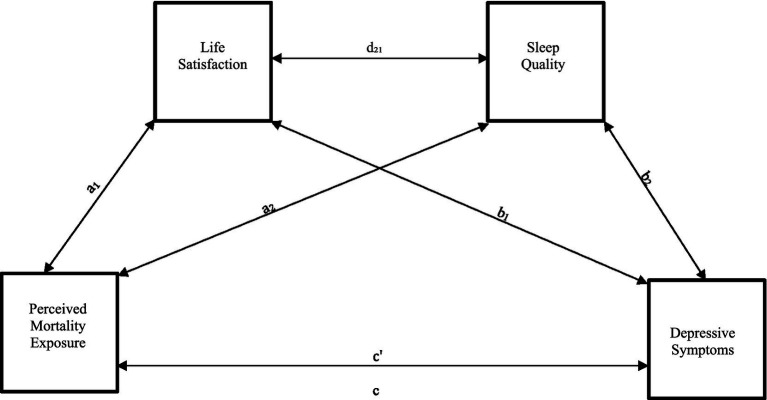
Serial mediation model (process Model 6) examining the direct and indirect effects of perceived mortality exposure on depressive symptoms through life satisfaction (SWLS) and sleep quality (PSQI).

A serial mediation analysis was conducted using Hayes’ PROCESS macro (Model 6; Figure 1) with 5,000 bootstrap samples to examine whether life satisfaction and sleep quality mediated the relationship between perceived mortality exposure and depressive symptoms, controlling for years of nursing experience and nationality. The total effect of perceived mortality exposure on depressive symptoms was significant (B = 1.67, SE = 0.70, t = 2.37, *p* = 0.019, 95% CI [0.28, 3.06]). However, when life satisfaction and sleep quality were included as mediators, the direct effect was no longer significant (B = 1.23, SE = 0.66, t = 1.86, *p* = 0.064, 95% CI [−0.07, 2.54]).

Examination of the indirect effects indicated that the total indirect effect was not significant (B = 0.44, BootSE = 0.32, 95% CI [−0.15, 1.11]). Similarly, the specific indirect effects through life satisfaction (B = 0.38, 95% CI [−0.05, 0.95]), sleep quality (B = 0.05, 95% CI [−0.33, 0.44]), and the sequential pathway (B = 0.001, 95% CI [−0.01, 0.02]) were all non-significant, as their confidence intervals included zero. These findings indicate that there was no evidence that life satisfaction or sleep quality mediated the relationship between perceived mortality exposure and depressive symptoms.

## 4 Discussion

The present study examined the relationships between perceived mortality exposure, life satisfaction, sleep quality, and depressive symptoms among nurses working in clinical settings. The findings revealed important associations among occupational, demographic, and psychological factors, providing insight into how perceived exposure to patient death relates to nurses’ well-being (4, 26).

The prevalence of poor sleep quality observed in this study was notably high. This finding should be interpreted in the context of the sample characteristics. The sample consisted of clinical nurses who are frequently exposed to shift work, demanding schedules, and emotionally challenging environments. The participants reported considerable difficulties across several sleep domains, including prolonged sleep latency, sleep disturbances, and daytime dysfunction, which collectively contributed to elevated PSQI scores. Nevertheless, the high prevalence may have reduced variability in sleep quality scores and limited the ability to detect mediation effects involving sleep quality.

The results indicated that perceived mortality exposure was significantly and positively associated with depressive symptoms, as well as with poorer sleep quality. These findings are consistent with previous studies examining the psychological consequences of exposure to patient death. Kurt and Ozsavran (1) reported that nurses working in paediatric intensive care units described patient deaths as emotionally distressing experiences that frequently resulted in feelings of sadness, helplessness, and emotional exhaustion. Similarly, Phillips et al. (3) identified professional grief as a common but often overlooked consequence of repeated exposure to patient loss among healthcare professionals. Ljubičić et al. (15) further reported that nurses working in palliative care settings experienced higher levels of professional stress and poorer mental health outcomes. Together, these findings support the present observation that greater exposure to patient death is associated with higher depressive symptoms and poorer sleep quality. Such exposure may increase emotional strain through heightened psychological arousal and ongoing cognitive engagement with patient loss (3). However, the magnitude of these associations was modest, indicating that perceived mortality exposure is one of several factors influencing nurses’ psychological health (27).

In contrast, perceived mortality exposure was not significantly associated with life satisfaction. This suggests that although exposure to patient death is related to specific aspects of psychological distress, it may not extend to broader evaluations of overall life satisfaction. One possible explanation is that nurses maintain a level of professional adaptation or emotional boundary-setting that protects their overall outlook on life despite exposure to challenging clinical experiences. Martins et al. (7) found that life satisfaction mediated the relationship between psychological distress and burnout among nurses during the COVID-19 pandemic, suggesting that life satisfaction may function as a protective psychological resource. Similarly, Petrosino et al. (9) reported that nurses with higher quality of life experienced lower sleep disturbance and lower intentions to leave critical care settings. These findings support the possibility that life satisfaction may be relatively stable and less directly affected by specific occupational stressors such as exposure to patient death. However, the lack of significant associations involving life satisfaction should be interpreted with caution. The Satisfaction with Life Scale measures a global cognitive evaluation of overall life circumstances rather than satisfaction within specific domains, such as work or professional experiences. Consequently, the broad nature of the measure may have limited its sensitivity to detect associations with occupational factors, including mortality exposure among nurses.

Nationality was found to be significantly associated with both life satisfaction and depressive symptoms, with Saudi nurses reporting lower life satisfaction and higher depressive symptoms compared to non-Saudi nurses. This pattern highlights the potential influence of sociocultural and occupational factors within the healthcare environment. Similar variations in psychological outcomes among nurses have been reported in other multicultural healthcare settings. For example, Auz et al. (4) found substantial variability in psychological responses among nurses during the COVID-19 pandemic, while Mohamed et al. (26) reported high rates of depression, anxiety, and stress among nurses, with workplace and social factors contributing to these outcomes. These findings suggest that occupational and sociocultural factor paediatrics may shape nurses’ psychological experiences differently across healthcare systems and populations.

Age and years of nursing experience were negatively associated with depressive symptoms, suggesting that more experienced nurses reported lower levels of depressive symptoms. This finding aligns with previous evidence suggesting that professional experience may protect against psychological distress. Saade et al. (27), in a systematic review of helping professions, reported that work-related risk factors and limited coping resources were consistently associated with depressive symptoms, whereas greater professional experience appeared to be associated with improved adaptation to occupational stressors. This may explain why older and more experienced nurses in the present study reported fewer depressive symptoms.

The regression analysis further supported the role of perceived mortality exposure as a significant predictor of depressive symptoms, even after controlling for demographic variables. This finding is consistent with the work of Ljubičić et al. (15), who reported that exposure to end-of-life care and patient death was associated with higher levels of professional stress and poorer mental health outcomes among nurses. However, the modest amount of explained variance in the current model suggests that perceived mortality exposure represents only one of several factors contributing to depressive symptoms. Other factors not examined in the present study, including burnout, moral distress, coping strategies, resilience, staffing adequacy, organisational support, and personal life stressors, may also contribute substantially to depressive symptoms among nurses.

The mediation analysis provided additional insight into the potential mechanisms underlying these relationships. Although perceived mortality exposure was initially associated with depressive symptoms, the indirect effects through life satisfaction and sleep quality were not statistically significant. This suggests that life satisfaction and sleep quality did not significantly explain the relationship between perceived mortality exposure and depressive symptoms in this sample. Furthermore, although the direct effect became non-significant after including the mediators, the absence of significant indirect effects suggests an inconclusive mediation pattern rather than evidence of mediation. Previous studies have identified several factors that may help explain nurses’ psychological well-being beyond sleep quality and life satisfaction. Wu et al. (6) found that stress overload and anxiety mediated the relationship between stress mindset and sleep quality, whereas Qin et al. (23) demonstrated that job satisfaction and sleep quality mediated the relationship between work stress and depression among healthcare workers. Collectively, these findings suggest that occupational and organisational variables may represent more proximal mechanisms linking workplace stressors to depressive symptoms than those examined in the present study (6, 9, 11, 25, 28).

The mediation findings should be interpreted with caution given the cross-sectional nature of the study. Although mediation analysis can be used to examine potential explanatory pathways, the simultaneous measurement of all study variables precludes establishing temporal ordering or causal relationships. Consequently, the observed associations between mortality exposure, life satisfaction, sleep quality, and depressive symptoms should be interpreted as correlational rather than causal. Longitudinal studies are needed to determine whether these variables operate through the pathways proposed in the present analysis.

### 4.1 Implications

The findings of this study have important implications for nursing practice, healthcare management, and policy, both locally and globally. First, the significant association between perceived mortality exposure and depressive symptoms underscores the need to recognize exposure to patient death as a meaningful occupational stressor within clinical environments. Healthcare organizations should move beyond viewing such exposure as an inevitable aspect of nursing and instead integrate structured psychological support into routine practice. This includes formal debriefing sessions following patient death, peer support programs, and facilitated reflective practice, which can help nurses process emotional experiences in a supportive context.

Second, although life satisfaction and sleep quality did not mediate the relationship between perceived mortality exposure and depressive symptoms, both variables remain clinically relevant indicators of nurses’ well-being. The strong association between life satisfaction and depressive symptoms suggests that interventions aimed at enhancing overall well-being—such as mentorship programs, professional development opportunities, and work–life balance initiatives—may indirectly contribute to improved mental health outcomes. Similarly, the high prevalence of poor sleep quality observed in this study highlights the need for organizational-level interventions targeting shift scheduling, workload management, and sleep hygiene education, even if sleep was not a significant predictor of depressive symptoms in the final model.

Third, the observed differences across nationality groups point to the importance of context-sensitive workforce strategies in multicultural healthcare systems. Healthcare organizations should consider tailored interventions that address the unique social and professional needs of diverse nursing populations, including culturally responsive support systems, equitable workload distribution, and inclusive workplace policies. Such strategies are particularly relevant in regions with a high proportion of expatriate nurses, where social integration and organizational belonging may influence psychological well-being.

At a broader level, these findings contribute to the growing recognition that psychosocial determinants of workforce stability are as critical as structural and financial factors. From a policy perspective, integrating mental health support into workforce planning frameworks may enhance retention, reduce burnout, and improve the quality of care. Globally, as healthcare systems continue to face workforce shortages and increasing clinical demands, prioritizing nurses’ psychological well-being is essential for sustaining a resilient and effective healthcare workforce.

### 4.2 Limitations

Several limitations should be considered when interpreting the findings. First, the cross-sectional design represents an important limitation of this study. Because all variables were measured at a single point in time, causal inferences cannot be made, and the direction of the observed relationships cannot be established. Although mediation analyses were conducted to explore potential mechanisms linking perceived mortality exposure with depressive symptoms, these findings should be interpreted as exploratory and associational. Future longitudinal or prospective studies are needed to examine temporal relationships among these variables.

Second, all variables were measured using self-report instruments, which may introduce response bias and common method variance. Although validated scales were used, future studies should incorporate multiple data sources or objective measures where possible. Additionally, although the study was guided by the Transactional Model of Stress and Coping, not all components of the model were directly assessed. Specifically, primary appraisal, secondary appraisal, coping strategies, and reappraisal processes were not measured. Therefore, the findings should be interpreted as examining selected variables that are conceptually consistent with the model rather than providing a comprehensive test of the theoretical framework.

Third, the study was conducted within a single healthcare organization in Saudi Arabia, which may limit the generalizability of the findings to other settings. Cultural, organizational, and workforce differences should be considered when interpreting the results.

Finally, the mediation model included only life satisfaction and sleep quality. Other important mechanisms—such as burnout, psychological distress, emotional exhaustion, or work-related support—were not examined and may better explain the relationship between perceived mortality exposure and depressive symptoms.

## 5 Conclusion

This study found that perceived mortality exposure was significantly associated with depressive symptoms among nurses, underscoring the psychological demands of repeated exposure to patient death in clinical settings. In contrast, the hypothesized mediating roles of life satisfaction and sleep quality were not supported, as neither variable significantly explained the relationship between perceived mortality exposure and depressive symptoms.

These findings suggest that the association between perceived mortality exposure and nurses’ mental health may operate through mechanisms other than general well-being or sleep-related processes, pointing to the potential role of factors such as coping strategies, emotional support, and organizational context.

Collectively, the results highlight the importance of addressing perceived mortality exposure as a distinct occupational stressor and reinforce the need for targeted, context-sensitive interventions to support nurses’ psychological well-being. Future research should adopt longitudinal and multi-method approaches to further clarify the pathways linking workplace stressors to mental health outcomes in nursing populations.

## Data Availability

The raw data supporting the conclusions of this article will be made available by the authors, without undue reservation.
